# Deaths from Fire: Are Trained Nurses' Uniform Dangerous?

**Published:** 1917-01-27

**Authors:** 


*To the Editor of* The Hospital.

### Deaths from Fire: Are Trained Nurses' Uniform Dangerous?

*To the Editor of* The Hospital.

Sir,—A few days ago a report appeared in the newspapers of a sad fatality by which a young girl lost her life through her clothing catching alight while standing in front of a fire.

As I had the grief of losing a much-loved daughter, of about the same age, under similar conditions, and, by a strange coincidence, almost on the same day of the month, three years ago, may I venture to give a warning in the hope that it may be the means of saving some other young life? In both cases the girls were engaged in hospital work, and, presumably, similarly clad in the usual print uniform; in each case the dress apparently ignited and burnt far more quickly than would be expected. My theory is that some substance of a highly inflammable nature, possibly petroleum, is used in the process of cleaning. Could not something be done by investigation into the materials used for this purpose to avert (if my theory is correct) the terrible danger to the many thousands of young lives of those who are at the present time wearing hospital uniforms. For obvious reasons I prefer that my name should not be published, but I enclose my card and subscribe myself "A Father."

